# GLI2-dependent c-MYC upregulation mediates resistance of pancreatic cancer cells to the BET bromodomain inhibitor JQ1

**DOI:** 10.1038/srep09489

**Published:** 2015-03-25

**Authors:** Krishan Kumar, Sania S. Raza, Lawrence M. Knab, Christina R. Chow, Benjamin Kwok, David J. Bentrem, Relja Popovic, Kazumi Ebine, Jonathan D. Licht, Hidayatullah G. Munshi

**Affiliations:** 1 Department of Medicine, Feinberg School of Medicine, Northwestern University, 303 E. Superior Ave, Chicago, IL 60611; 2 Department of Surgery, Feinberg School of Medicine, Northwestern University, 676 N. St. Clair St, Chicago, IL 60611; 3 Jesse Brown VA Medical Center, 820 S. Damen Ave, Chicago, IL 60612; 4 The Robert H. Lurie Comprehensive Cancer Center of Northwestern University, 303 E. Superior Ave, Chicago, IL 60611

## Abstract

JQ1 and I-BET151 are selective inhibitors of BET bromodomain proteins that have efficacy against a number of different cancers. Since the effectiveness of targeted therapies is often limited by development of resistance, we examined whether it was possible for cancer cells to develop resistance to the BET inhibitor JQ1. Here we show that pancreatic cancer cells developing resistance to JQ1 demonstrate cross-resistance to I-BET151 and insensitivity to BRD4 downregulation. The resistant cells maintain expression of c-MYC, increase expression of JQ1-target genes FOSL1 and HMGA2, and demonstrate evidence of epithelial-mesenchymal transition (EMT). However, reverting EMT fails to sensitize the resistant cells to JQ1 treatment. Importantly, the JQ1-resistant cells remain dependent on c-MYC that now becomes co-regulated by high levels of GLI2. Furthermore, downregulating GLI2 re-sensitizes the resistant cells to JQ1. Overall, these results identify a mechanism by which cancer cells develop resistance to BET inhibitors.

There has been increasing interest in targeting the bromodomain (BRD) and extra terminal domain (BET) family of proteins in a number of different cancer types^1^,^2^,^3^,^4^,^5^. BET proteins – BRD2-4 and BRDT – are important ‘reader' molecules that bind to acetylated histones to regulate transcription of genes involved in growth, fibrosis, and inflammation^1^,^2^,^3^,^4^,^5^,^6^. JQ1 and I-BET151^1^,^7^, the two most studied selective inhibitors of BET proteins, have been shown to inhibit growth of blood cancers and solid tumors *in vitro* and in xenograft models^1^,^3^,^5^,^8^,^9^,^10^,^11^. These compounds potently inhibit growth of leukemia, lymphoma and neuroblastoma cell lines through repression of MYC and its downstream transcriptional targets^2^,^4^,^5^,^12^. However, the effect of JQ1 on growth of lung cancer cells was found instead to be through repression of FOS-like antigen 1 (FOSL1)^3^. We found that BET inhibitors decrease growth of pancreatic cancer cells through repression of both c-MYC and FOSL1^13^. Additionally, the BET inhibitors repress high mobility group A2 (HMGA2)^13^, an architectural protein that regulates chromatin structure^14^,^15^, and which we previously showed to contribute to chemotherapy resistance^16^,^17^.

Unfortunately, the effectiveness of targeted therapies is often limited by development of resistance^18^. Overexpression of the target protein or a mutation resulting in decreased binding of the small molecule inhibitor was shown to mediate resistance to targeted therapies^18^. Cells may also activate alternative pathways to bypass the effects of a small molecule inhibitor^18^. Additionally, cells may demonstrate epigenetic changes to overcome the effects of target inhibition. For example, cells may undergo epithelial-mesenchymal transition (EMT), which has been shown to mediate resistance to both targeted therapies and chemotherapy^19^,^20^. EMT is induced by a number of transcription factors (e.g., Snail, Slug, ZEB1) and microRNAs that repress E-cadherin and upregulate mesenchymal markers^21^,^22^.

In this report, we examined whether it was possible for pancreatic cancer cells to develop resistance to the BET inhibitor JQ1. We show that the CD18 pancreatic cancer cells developing resistance to JQ1 are resistant to BRD4 knockdown and maintain or increase expression of JQ1-target genes. The JQ1-resistant cells demonstrate decreased cell-cell and cell-matrix adhesion associated with increased ZEB1 expression. Although ZEB1 siRNA restores cell-cell and cell-matrix adhesion in the JQ1-resistant cells, ZEB1 siRNA fails to sensitize resistant cells to JQ1 treatment. Importantly, the JQ1-resistant cells remain dependent on c-MYC that now becomes co-regulated by high levels of GLI2. Significantly, downregulating GLI2 re-sensitizes the resistant cells to JQ1. Overall, these results identify a mechanism by which cancer cells develop resistance to BET inhibitors.

## Results

### JQ1-resistant pancreatic cancer cells are resistant to BRD4 knockdown and demonstrate rebound increase in JQ1-target genes

**Figure 1:**
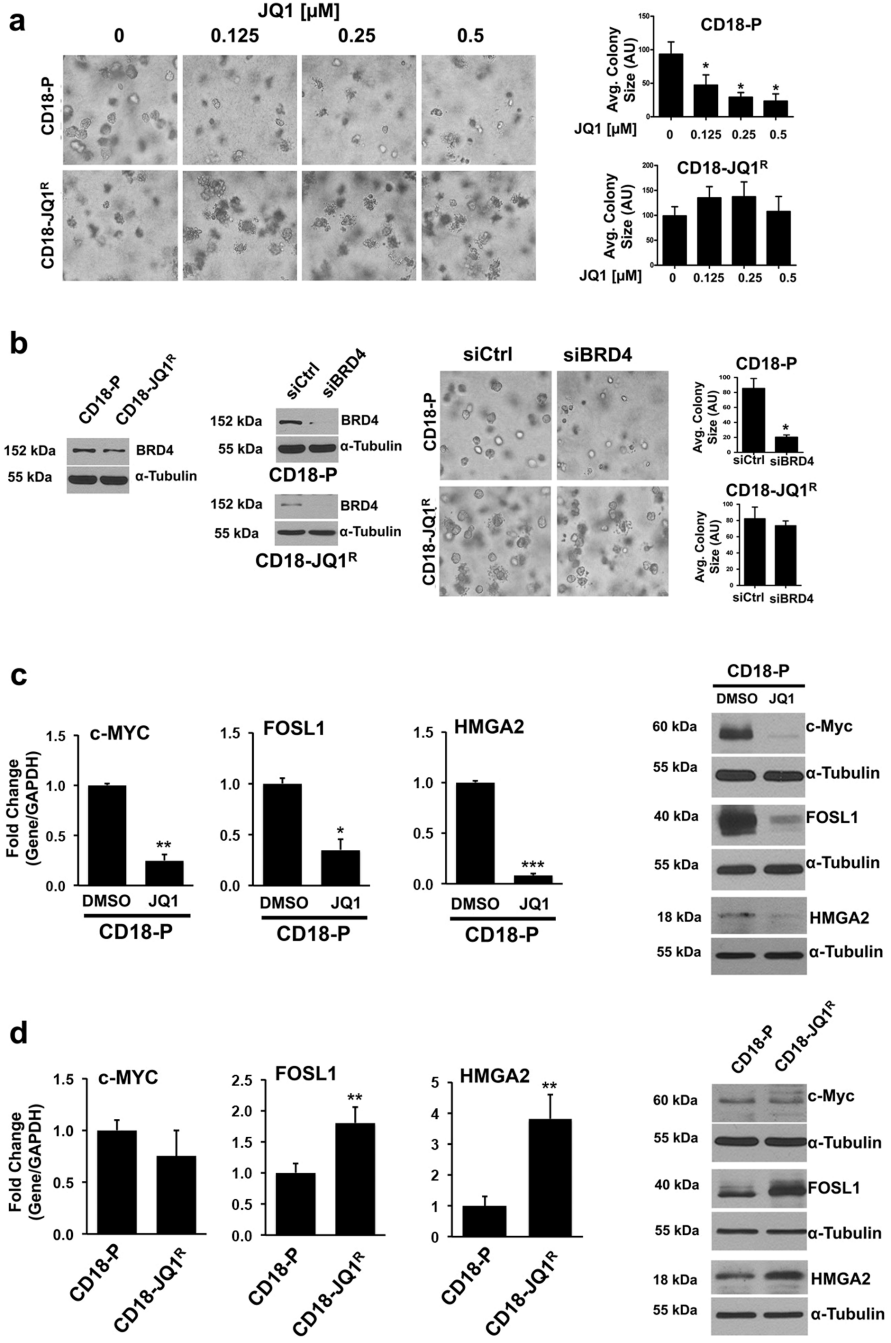
JQ1-resistant pancreatic cancer cells are resistant to BRD4 knockdown and demonstrate rebound increase in JQ1-target genes. (a) Parental (CD18-P) and JQ1-resistant (CD18-JQ1^R^) pancreatic cancer cells were grown in three-dimensional collagen gels and fresh serum-containing medium supplemented with DMSO or JQ1 was added every other day for 4 days. The effect on colony size was examined by phase contrast microscopy, and size of the individual colonies measured. (b) Lysates from CD18-P and CD18-JQ1^R^ cells growing on tissue culture plastic were analyzed for BRD4 by Western blotting. CD18-P and CD18- JQ1^R^ cells were transfected with control siRNA (siCtrl) or BRD4-specific siRNAs (siBRD4), allowed to recover for 48 hours, and then embedded in collagen gels. The specific knockdown of BRD4 was determined by Western blotting. The effect on colony size in three-dimensional collagen was examined by phase contrast microscopy, and size of the individual colonies was measured. (c) CD18-P cells were grown in three-dimensional collagen gels in the presence of DMSO or JQ1 (0.5 μM) for 48 hours. The effect on c-MYC, FOSL1 and HMGA2 expression was analyzed by qRT-PCR and Western blotting. (d) CD18-P and CD18-JQ1^R^ cells were grown in three-dimensional collagen gels for 48 hours, and the effect on c-MYC, FOSL1 and HMGA2 expression was analyzed by qRT-PCR and Western blotting. *, p < 0.05; **, p < 0.01; ***, p <0.001 relative to control samples. The results are representative of at least four independent experiments. See also Figure S1.

Recently, we demonstrated that BET inhibitors are effective against pancreatic cancer cells growing in three-dimensional collagen (Fig. 1a)^13^. Since cancer cells can eventually develop resistance to therapeutic agents^18^, we treated CD18 pancreatic cancer cells with increasing concentrations of JQ1 over a prolonged period of time to generate CD18 cells resistant to JQ1 (CD18-JQ1^R^). These cells, in contrast to parental CD18 cells (CD18-P), continued to grow in 3D collagen in the presence of increasing concentrations of JQ1 (Fig. 1a). Significantly, CD18-JQ1^R^ cells were also resistant to the structurally-related BET inhibitor I-BET151 (Supplementary Fig. S1). Since the effects of JQ1 in CD18 cells are primarily mediated by inhibition of BRD4^13^, we examined whether there was increased expression of BRD4 protein in CD18-JQ1^R^. The levels of BRD4 protein were in fact *lower* in CD18-JQ1^R^ cells (Fig. 1b). Moreover, while BRD4 siRNA repressed growth of CD18-P cells, CD18-JQ1^R^ cells were resistant to the effects of BRD4 knockdown (Fig. 1b). We next examined the levels of JQ1-target genes in CD18-P and CD18-JQ1^R^ cells. As shown previously^13^, expression of c-MYC, FOSL1 and HMGA2 was repressed following acute treatment of CD18-P cells with JQ1 (Fig. 1c). In contrast, CD18-JQ1^R^ cells treated continuously with JQ1 demonstrate minimally decreased levels of c-MYC and in fact have increased FOSL1 and HMGA2 levels (Fig. 1d).

### JQ1-resistant pancreatic cancer cells demonstrate decreased cell-cell and cell-matrix adhesion associated with increased ZEB1 expression, but ZEB1 siRNA fails to sensitize resistant cells to JQ1 treatment

**Figure 2:**
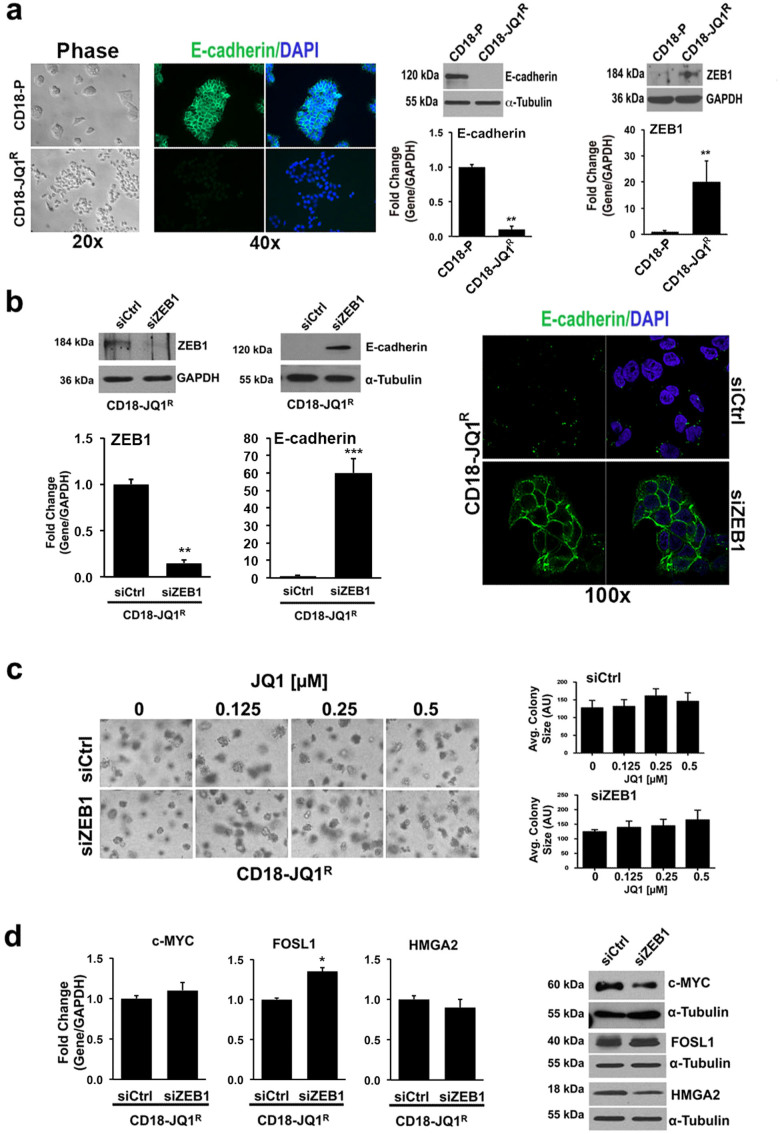
JQ1-resistant pancreatic cancer cells demonstrate decreased cell-cell adhesion associated with increased ZEB1 expression, but ZEB1 siRNA fails to sensitize resistant cells to JQ1 treatment. (a) CD18-P and CD18-JQ1^R^ growing on tissue culture plastic were examined by phase microscopy. E-cadherin localization in CD18-P and CD18-JQ1^R^ cells was analyzed by immunofluorescence using DAPI to counterstain nuclei. Lysates from CD18-P and CD18-JQ1^R^ cells were analyzed for E-cadherin and ZEB1 expression by qRT-PCR and Western blotting. (b) CD18-JQ1^R^ cells growing on tissue culture plastic were transfected with control siRNA (siCtrl) or ZEB1-specific siRNA (siZEB1) for 72 hours. The specific knockdown of ZEB1 and the effect on E-cadherin expression was determined by qRT-PCR and Western blotting. Effect on cell morphology was analyzed by phase microscopy and E-cadherin cellular localization was analyzed by immunofluorescence using DAPI to counterstain nuclei. (c, d) CD18-JQ1^R^ cells plated onto tissue culture plastic were transfected with siCtrl or siZEB1 for 48 hours, embedded in collagen gels, and treated with JQ1 every other day for 4 days. The effect on colony size in three-dimensional collagen was examined by phase contrast microscopy, and size of the individual colonies measured. The effect on c-MYC, FOSL1 and HMGA2 expression was analyzed by qRT-PCR and by Western blotting. The results are representative of at least three independent experiments. *, p < 0.05; **, p < 0.01; ***, p <0.001 relative to control samples. See also Figure S2.

While CD18-P cells grow in cohesive clumps, CD18-JQ1^R^ cells grow primarily as single cells and failed to express E-cadherin (Fig. 2a), suggesting that JQ1 resistance was accompanied by an epithelial-mesenchymal transition (EMT). Although there was no significant change in the levels of *Snail* and *Slug,* both regulators of EMT^21^,^22^, in CD18-JQ1^R^ cells compared to CD18-P cells (data not shown), CD18-JQ1^R^ cells express increased ZEB1 mRNA and protein levels (Fig. 2a). The CD18-JQ1^R^ cells also express significantly lower levels of *miR-200a* and *miR-200c* (data not shown)*,* microRNAs that target *ZEB1*^23^.

As activation of integrin signaling can contribute to resistance to anti-tumor therapies^24^, we examined whether there was increased integrin activation and cell adhesion to collagen in CD18-JQ1^R^ cells compared to CD18-P cells. However, CD18-JQ1^R^ cells have in fact *decreased* adhesion to collagen matrix (Supplementary Fig. S2a). CD18-JQ1^R^ cells also showed reduced cell-surface expression of the collagen-binding α2-integrin without significantly affecting cell-surface levels of β1-integrin (Supplementary Fig. S2b). Moreover, CD18-JQ1^R^ cells have significant reduction in FAK phosphorylation compared to CD18-P cells (Supplementary Fig. S2c). These results indicate that CD18-JQ1^R^ cells in fact have *decreased* signaling downstream of collagen-binding integrins.

Since it was shown that reverting EMT could restore sensitivity of lung cancer cells to targeted therapy^20^, we examined the effect of ZEB1 siRNA on the response of CD18-JQ1^R^ cells to JQ1. Transfection with ZEB1 siRNA restored E-cadherin levels and enhanced cell-cell adhesion (Fig. 2b). ZEB1 siRNA also restored adhesion of CD18-JQ1^R^ cells to collagen matrix and increased expression of α2-integrin (Supplementary Fig. S2d, e). However, despite reversing these aspects of the EMT, ZEB1 siRNA failed to re-sensitize CD18-JQ1^R^ cells to JQ1 treatment (Fig. 2c). Moreover, ZEB1 siRNA had minimal effects on c-MYC, FOSL1 and HMGA2 mRNA and protein levels in CD18-JQ1^R^ cells (Fig. 2d).

### JQ1-resistant pancreatic cancer cells demonstrate increased GLI2 expression

**Figure 3:**
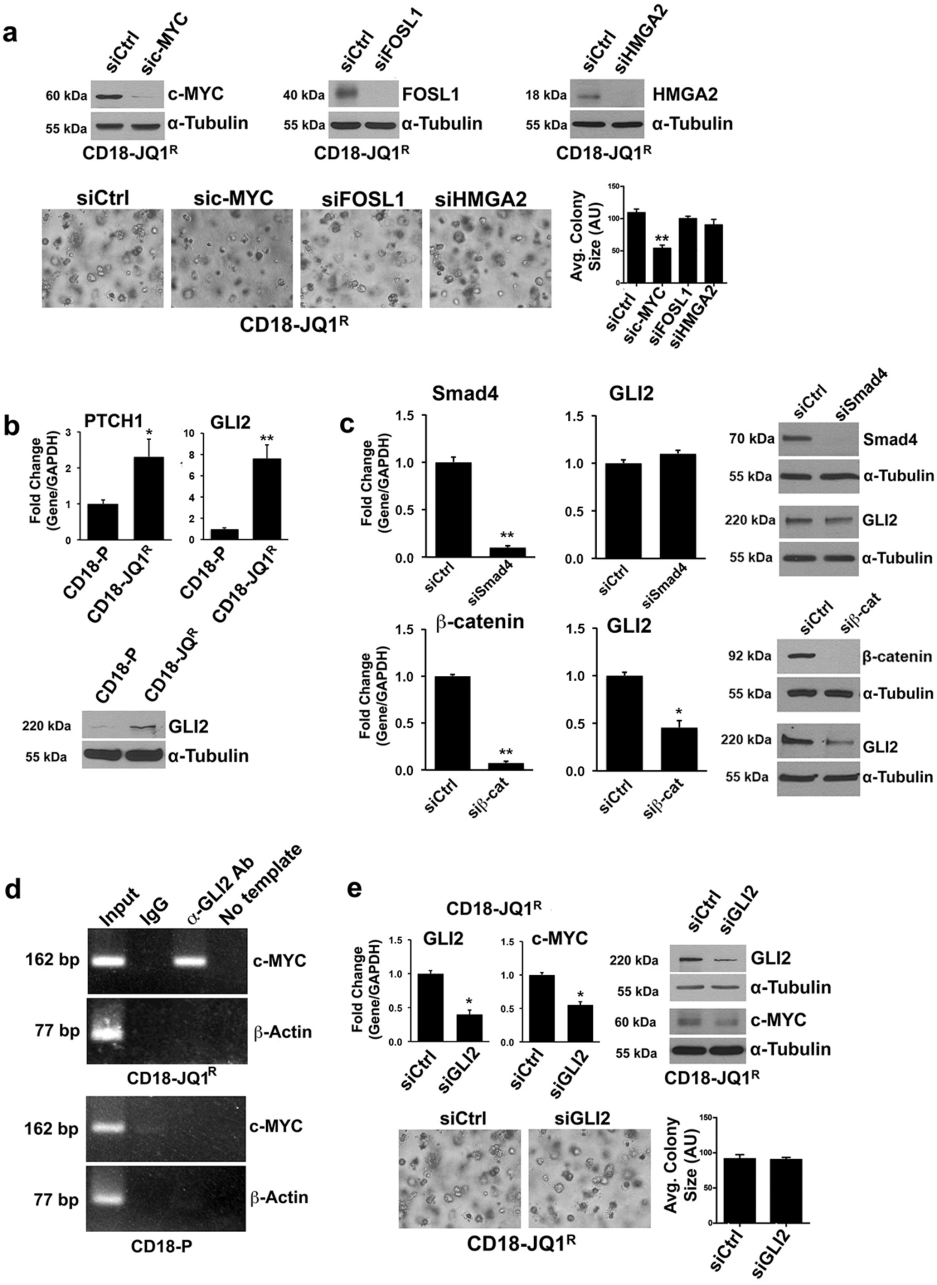
JQ1-resistant pancreatic cancer cells demonstrate increased GLI2 expression. (a) CD18-JQ1^R^ cells were transfected with control siRNA (siCtrl), c-MYC-specific siRNA (sic-MYC), FOSL1-specific siRNA (siFOSL1) or HMGA2-specific siRNA (siHMGA2). The cells were allowed to recover for 48 hours, and then embedded in collagen gels. The specific knockdown of individual proteins was determined by Western blotting. The effect on colony size in three-dimensional collagen was examined by phase contrast microscopy, and size of the individual colonies measured. (b) CD18-P and CD18-JQ1^R^ cells were analyzed for Patched1 (PTCH1) and GLI2 expression by qRT-PCR and GLI2 protein levels by Western blotting. (c) CD18-JQ1^R^ cells were transfected with siCtrl, Smad4-specific siRNA (siSmad4) or β-catenin-specific siRNA (siβ-cat) and the effect on GLI2 mRNA and protein expression was determined. (d) Chromatin immunoprecipitation was performed across MYC locus in CD18-P and CD18-JQ1^R^ cells with control IgG antibody or anti-GLI2 antibody. Purified DNA was then analyzed by PCR using primers specific for c-MYC locus, and the PCR products visualized on 2% agarose gels. As additional control, the purified DNA was also analyzed for β-actin by PCR (e) CD18-JQ1^R^ cells were transfected with siCtrl or GLI2-specific siRNA (siGLI2) for 48 hours, and then embedded in three-dimensional collagen for 24 hours. The specific knockdown of GLI2 was determined by qRT-PCR and Western blotting. The effect of siGLI2 on c-MYC expression was analyzed by qRT-PCR and Western blotting. The effect of siGLI2 on colony size in three-dimensional collagen was examined by phase contrast microscopy, and size of the individual colonies was measured. *, p < 0.05; **, p < 0.01 relative to control samples. The results are representative of at least three independent experiments.

In order to characterize the resistance mechanism, we evaluated the extent to which CD18-JQ1^R^ cells remained dependent on c-MYC, FOSL1 or HMGA2 for growth in 3D collagen using siRNA to knockdown each protein. CD18-JQ1^R^ cells require c-MYC, but not FOSL1 or HMGA2, for growth in 3D collagen (Fig. 3a). Thus, we screened additional JQ1-independent pathways that may regulate c-MYC levels in CD18-JQ1^R^ cells.

The hedgehog (Hh) signaling pathway, which is both an early and a late mediator of pancreatic cancer tumorigenesis^25^,^26^, can regulate c-MYC expression^27^. Initially, we evaluated whether there was alteration in Hh signaling by examining relative expression of *Patched1, Patched2, GLI1* and *GLI2* in CD18-P and CD18-JQ1^R^ cells. As CD18-P and CD18-JQ1^R^ cells do not express *GLI1* or *Patched2* mRNA (data not shown), we compared the relative levels of *Patched1* and *GLI2* in CD18-P and CD18-JQ1^R^ cells. *Patched1* levels were modestly higher in CD18-JQ1^R^ than in CD18-P cells; however, *GLI2* mRNA was ~7-fold higher in CD18-JQ1^R^ than in CD18-P cells (Fig. 3b). There was also increased expression of GLI2 protein in CD18-JQ1^R^ cells (Fig. 3b). Since the GLI2 promoter has SMAD and lymphoid enhancer factor/T cell factor binding sites^28^, we examined the role of Smad and β-catenin signaling in mediating GLI2 expression in CD18-JQ1^R^ cells. Down-regulating β-catenin, but not Smad4, attenuated GLI2 expression in CD18-JQ1^R^ cells (Fig. 3c).

We next evaluated the extent to which GLI2 regulated c-MYC levels in CD18-JQ1^R^ cells. Initially, we conducted chromatin immunoprecipitation (ChIP) to evaluate whether GLI2 bound to c-MYC promoter in CD18-JQ1^R^ cells. ChIP with anti-GLI2 antibody demonstrated binding of GLI2 to the c-MYC promoter in CD18-JQ1^R^ cells, but not in CD18-P cells (Fig. 3d). Additionally, GLI2 knockdown resulted in ~50% reduction in c-MYC levels in CD18-JQ1^R^ cells (Fig. 3e); however, GLI2 siRNA failed to block growth of CD18-JQ1^R^ cells in 3D collagen. These results indicate that partial repression of c-MYC by GLI2 siRNA may not be sufficient to affect growth of CD18-JQ1^R^ cells.

### Targeting GLI2 restores sensitivity of JQ1-resistant cells to BET inhibitors

**Figure 4:**
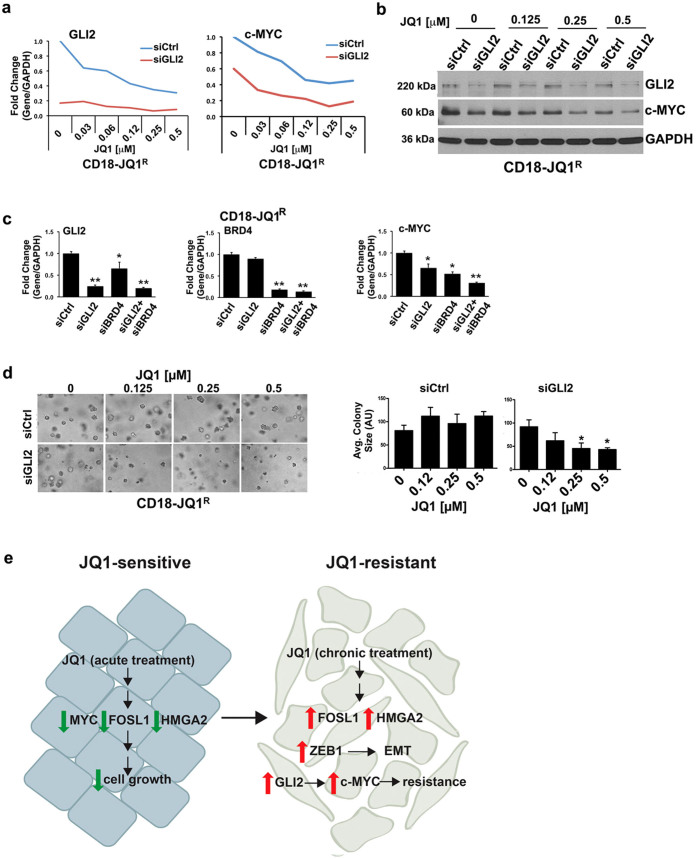
Targeting GLI2 restores sensitivity of JQ1-resistant cells to BET inhibitors. (a, b) CD18-JQ1^R^ cells growing on tissue culture plastic were transfected with siCtrl or GLI2-specific siRNA (siGLI2) for 48 hours, embedded in collagen gels and treated with JQ1 for 24 hours. The effect on GLI2 and c-MYC expression was analyzed by qRT-PCR and Western blotting. (c) CD18-JQ1^R^ cells were transfected with siCtrl, siGLI2, BRD4-specific siRNA (siBRD4), or a combination of siGLI2 and siBRD4 for 48 hours, and then embedded in three-dimensional collagen for 24 hours. The effect on GLI2, BRD4 and c-MYC was determined by qRT-PCR. (d) CD18-JQ1^R^ cells growing on tissue culture plastic were transfected with siCtrl or siGLI2 for 48 hours, embedded in collagen gels and treated with JQ1 for 4 days. The effect on colony size in three-dimensional collagen was examined by phase contrast microscopy, and size of the individual colonies measured. *, p < 0.05; **, p < 0.01 relative to control samples. The results are representative of at least three independent experiments. (e) Model: PDAC cells developing resistance maintain or increase expression of JQ1-target genes c-MYC, FOSL1 and HMGA2, and also demonstrate EMT associated with ZEB1 expression. Significantly, JQ1-resistant cells remain dependent for growth on c-MYC, now co-regulated by GLI2, and that targeting GLI2 restores JQ1 sensitivity.

We next examined whether combination of JQ1 and GLI2 siRNA would result in further repression of c-MYC and thereby limit growth of CD18-JQ1^R^ cells in 3D collagen. In agreement with a recent paper demonstrating that GLI2 is a target of JQ1 in medulloblastoma cells^29^, treatment of CD18-JQ1^R^ cells with JQ1 resulted in partial repression of GLI2. Although GLI2 siRNA alone repressed *c-MYC* mRNA by ~40%, combination of JQ1 and GLI2 siRNA resulted in ~80% inhibition of *c-MYC* mRNA levels starting at the 0.125 μM JQ1 (Fig. 4a). JQ1 also enhanced the repressive effect of siGLI2 on c-MYC protein levels in CD18-JQ1^R^ cells (Fig. 4b). Additionally, co-transfection of BRD4 siRNA with GLI2 siRNA led to ~75% inhibition of *c-MYC* mRNA levels in CD18-JQ1^R^ (Fig. 4c). These results indicate that the c-MYC levels in CD18-JQ1^R^ cells are now co-regulated by both BRD4 and GLI2. Significantly, GLI2 siRNA restored sensitivity of CD18-JQ1^R^ cells to growth inhibition by JQ1 (Fig. 4d).

## Discussion

Since epigenetic changes have been implicated in every aspect of cancer development and progression, including response to therapy and recurrence^30^,^31^, there is an increasing interest in targeting these changes in solid tumors and blood cancers^32^,^33^,^34^,^35^,^36^,^37^,^38^. We have previously reported that human pancreatic tumors demonstrate increased histone acetylation in areas of fibrosis and that targeting histone acetyltransferases can sensitize pancreatic cancer cells to chemotherapy^16^. We recently reported that targeting readers of acetylation with BET inhibitors can also limit the growth of cancer cells in the collagen microenvironment^13^. In this report, we show that pancreatic cancer cells that develop resistance to BET inhibitors upregulate GLI2 and that targeting GLI2 re-sensitizes the pancreatic cancer cells to BET inhibitors.

Significantly, GLI levels are modulated in many forms of resistant cancer. For example, GLI levels are increased in cancer cells isolated from chemo-resistant ovarian cancer cells compared to matched primary tumors^39^. Accordingly, inhibition of GLI2 re-sensitizes ovarian tumors obtained from recurrent platinum-resistant patients to cisplatin^39^,^40^, and also reverses taxane resistance in ovarian cancer^41^. Mechanistically, GLI proteins can upregulate not only BCL2^42^,^43^, a key molecule involved in the suppression of the intrinsic apoptotic pathway, but also mediate resistance to extrinsic apoptotic pathway by increasing cFLIP expression^44^. In JQ1-resistant cells, we found that GLI2 along with BRD4 maintains expression of c-MYC, which is required for continued growth of these cells in the collagen microenvironment. In support of our findings, ectopic GLI2 expression was recently demonstrated to rescue growth inhibition by JQ1 in Hh-driven medulloblastoma cells^29^, suggesting the presence of GLI2-responsive promoters that do not require BRD4 for transactivation. However, our results suggest that c-MYC promoter is dependent on both GLI2 and BRD4 for transactivation in the JQ1-resistant cells.

We also show that the JQ1-resistant cells demonstrate evidence of EMT, which is also associated with resistance to therapy in pancreatic cancer cells^45^,^46^,^47^. Expression profiling of chemo-resistant cell lines have shown a strong association between EMT and chemotherapy resistance^47^. The EMT transcription factor ZEB1 is upregulated in chemotherapy resistant cell lines and silencing ZEB1 with siRNA reverses EMT and restores chemo-sensitivity^47^. EMT also plays a role in modulating resistance to targeted biologic therapies as well. Cells that express high levels of Snail or ZEB1 demonstrate significantly decreased growth inhibition in response to treatment with the EGFR inhibitor erlotinib compared to cells with an epithelial phenotype^48^. Despite the importance of ZEB1 and EMT in mediating resistance to chemotherapy and targeted therapies, we show that reversal of EMT through downregulation of ZEB1 failed to sensitize the JQ1-resistant cells to BET inhibitors.

Integrin signaling can also mediate resistance to chemotherapy and targeted therapies. Increased adhesion to ECM and activation of β1-integrin inhibits apoptosis in response to chemotherapy in breast and lung cancer cells^24^,^49^. Increased integrin activation also mediates resistance to anti-HER2 targeted therapy in breast cancer cells and to EGFR inhibitors in lung cancer cells^50^,^51^. We have previously shown that 3D collagen I contributes to resistance to gemcitabine in pancreatic cancer cells^17^. Others showed that increased adhesion to laminin and collagen IV also decreases sensitivity of pancreatic cancer cells to gemcitabine through FAK activation^52^. Despite the importance of integrin signaling in mediating resistance to chemotherapy and targeted therapies, we found that JQ1-resistant cells have *decreased* integrin expression and signaling, indicating that this mechanism was not the cause of drug resistance.

Overall, we demonstrate that cancer cells developing resistance to BET bromodomain inhibitors demonstrate EMT, decreased integrin signaling, and activation of the hedgehog pathway (Fig. 4e, Model). Although EMT does not mediate resistance to BET inhibitors, blocking the expression of GLI2 restores JQ1 sensitivity. Our findings demonstrate that a better understanding of the mechanism of resistance to BET inhibitors may allow for identification of additional therapeutic targets that extend the efficacy of BET inhibitors.

## Methods

### Reagents

Antibodies against c-MYC, FOSL1 and GLI2 were purchased from Cell Signaling, HMGA2 antibody was from Biocheck Inc, while vimentin and BRD4 antibodies were from Abcam. ZEB1, α-tubulin and total FAK antibodies were obtained from Santa Cruz, while E-cadherin antibody was from Invitrogen. pFAK(Y397) antibody was from BD Transduction laboratories, while GAPDH and α2- and β1-integrin antibodies were from Millipore. Secondary antibodies were purchased from Sigma. The EZ-Chip and EZ-Zyme Chromatin Prep kits were from Millipore. The anti-GLI2 rabbit antibody for ChIP assay was purchased from Abcam, while the control IgG rabbit antibody was from Cell Signaling. BET inhibitor JQ1 was obtained from BPS Bioscience, while I-BET151 was acquired from Tocris Bioscience. BRD4, c-MYC, FOSL1, ZEB1 and GLI2 siRNAs were purchased from Life Technologies.

### Cell culture

CD18/HPAF-II cells were obtained from American Type Culture Collection (ATCC; Manassas, VA) in 2008. Cells were maintained in DMEM containing 10% FBS and antibiotics (100 U/ml Penicillin and 100 µg/ml Streptomycin). JQ1-resistant CD18 (CD18-JQ1^R^) cells were generated by treating parental CD18 (CD18-P) cells with increasing concentrations of JQ1 over a period of 3 months. The surviving cells were maintained in 2.5 μM JQ1. The CD18-P and CD18-JQ1^R^ cells were authenticated by STR profiling at the Johns Hopkins Genetic Resources Core Facility in October, 2013.

### Transfection

Cells were transfected with siRNA against BRD4, c-MYC, FOSL1, GLI2, HMGA2, ZEB1 or control siRNA using Lipofectamine RNAimax (Invitrogen) according to manufacturer's instructions before plating into collagen.

### Embedding and examination of cells in three-dimensional type I collagen gels

Collagen mixture (2 mg/mL) was made by adding the appropriate volumes of sterile water, 10X DMEM and NaOH and kept on ice until needed^53^. Cells were then suspended in the collagen solution and allowed to gel at 37°C. For RNA extraction, the gel containing cells was processed using RNeasy extraction kit (Qiagen) and then processed for qRT-PCR analysis. For morphological examination of cells, cell colonies in three-dimensional collagen were examined using a Zeiss Axiovert 40 CFL microscope and pictures taken with a Nikon Coolpix 4500 camera^53^. The relative size of individual colonies was measured using Photoshop.

### Quantitative Real Time-PCR analysis

Quantitative mRNA and microRNA expression was performed with gene specific Taqman probes, TaqMan Universal PCR Master Mix and the 7500 Fast Real-time PCR System from Applied Biosystems. The data were then quantified with the comparative C_T_ method for relative gene expression.

### Immunoblotting

Immunoblotting for ZEB1, E-cadherin, vimentin, BRD4, c-MYC, FOSL1, HMGA2, GLI2, GAPDH and α-tubulin was done as previously described^53^.

### Adhesion assay

Equal numbers of CD18-P and CD18-JQ1^R^ cells were seeded onto tissue culture plates pre-coated with type I collagen. Cells were allowed to adhere at 37 °C for 10 minutes, washed once with PBS, and then fixed and stained^54^. Cells were imaged using a Zeiss microscope and photographed using a Nikon camera.

### Flow Cytometric Analysis

CD18-P and CD18-JQ1^R^ cells in suspension were incubated with anti α2- or β1-integrin antibody and then stained with secondary antibody conjugated to Alexa Fluor 488 prior to analysis with Summit Software 4.3 on a Beckman Coulter fluorescence-activated cell sorter^55^.

### Immunofluorescence

CD18-P and CD18-JQ1^R^ cells plated overnight onto collagen-coated glass coverslips were fixed and incubated with E-cadherin antibody followed by secondary antibody conjugated to Alexa Fluor 488. The cells were stained with DAPI after which the cells were washed, mounted, and observed using a Zeiss Axiovert 200 microscope.

### Chromatin Immunoprecipitation

CD18-P and CD18-JQ1^R^ cells were treated with formaldehyde to create DNA-protein cross-links. Chromatin fragments were prepared using EZ-Zyme Chromatin Prep kit and then ChIP performed using the EZ ChIP kit and anti-GLI2 antibody or control IgG antibody. Purified DNA was then analyzed by PCR using primers specific for c-MYC locus^56^, and the PCR products visualized on a 2%-agarose gel.

### Statistical analysis

All statistical analyses were done using GraphPad Instat using a two-tailed *t*-test analysis. Error bars represent standard deviation.

## Supplementary Material

Supplementary Information 2 supplementary figures
