# Supplementary material for: GLI2-dependent c-MYC upregulation mediates resistance of pancreatic cancer cells to the BET bromodomain inhibitor JQ1
Source: Sci Rep. 2015 Mar 25;5:9489. doi: 10.1038/srep09489 (PMC4452877; doi:10.1038/srep09489)
Supplement: Supplementary Information — 2 supplementary figures [file srep09489-s1.pdf]

GLI2-dependent c-MYC upregulation mediates resistance of pancreatic cancer cells to the BET bromodomain inhibitor JQ1

Krishan Kumar[1,3,5], Sania S. Raza[1,5], Lawrence M. Knab[2,5], Christina R. Chow[1,4], Benjamin Kwok[1], David J. Bentrem[2,3,4], Relja Popovic[1], Kazumi Ebine[1,3], Jonathan D. Licht[1,4], Hidayatullah G. Munshi[1,3,4,*]

Departments of [1]Medicine and [2]Surgery, Feinberg School of Medicine, Northwestern University; [3]Jesse Brown VA Medical Center; and [4]The Robert H. Lurie Comprehensive Cancer Center of Northwestern University, Chicago, IL 60611.

[5]Co-first author

Supplementary Material

SUPPLEMENTARY FIGURE LEGENDS

Figure S1: **JQ1-resistant pancreatic cancer cells are resistant to the BET inhibitor I-BET151.** Parental (CD18-P) and JQ1-resistant (CD18-JQ1^{R}) pancreatic cancer cells were grown in three-dimensional collagen gels and fresh serum-containing medium supplemented with DMSO or I-BET151 was added every other day for 4 days. The effect on colony size was examined by phase contrast microscopy, and size of the individual colonies measured. *, $p < 0.05$. The results are representative of three independent experiments.

Figure S2: **JQ1-resistant pancreatic cancer cells demonstrate decreased cell-matrix adhesion associated with increased ZEB1 expression.** (**a**) CD18-P and CD18-JQ1^{R} cells were seeded onto collagen I (Col I)-coated tissue culture plates and allowed to adhere at 37°C for 10 minutes. Adherent cells were photographed and counted. (**b**) CD18-P and CD18-JQ1^{R} cells were analyzed for collagen-binding α2- and β1-integrin expression by FACS analysis. (**c**) CD18-P and CD18-JQ1^{R} cells plated for 24 hours onto collagen-coated tissue culture plates were analyzed for pFAK(Y397) and total FAK by Western blotting. (**d, e**) CD18-JQ1^{R} cells growing on tissue culture plastic were transfected with control siRNA (siCtrl) or ZEB1-specific siRNA (siZEB1) for 72 hours. The transfected cells were re-plated onto collagen I (Col I)-coated tissue culture plates and allowed to adhere at 37°C for 10 minutes. Adherent cells were photographed and counted. The effect of ZEB1 knockdown on collagen-binding α2- and β1-integrin expression was evaluated by FACS analysis. *, $p < 0.05$. The results are representative of three independent experiments.

Figure S1

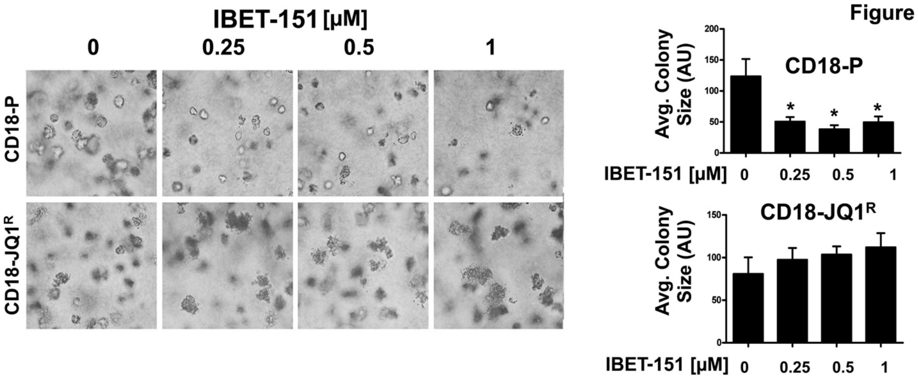

IBET-151 [μM]
0
0.25
0.5
1
CD18-P
CD18-JQ1R
CD18-P
Avg. Colony Size (AU)
200
150
100
50
0
*
*
*
IBET-151 [μM] 0 0.25 0.5 1
CD18-JQ1R
Avg. Colony Size (AU)
150
100
50
0
IBET-151 [μM] 0 0.25 0.5 1

Figure S2

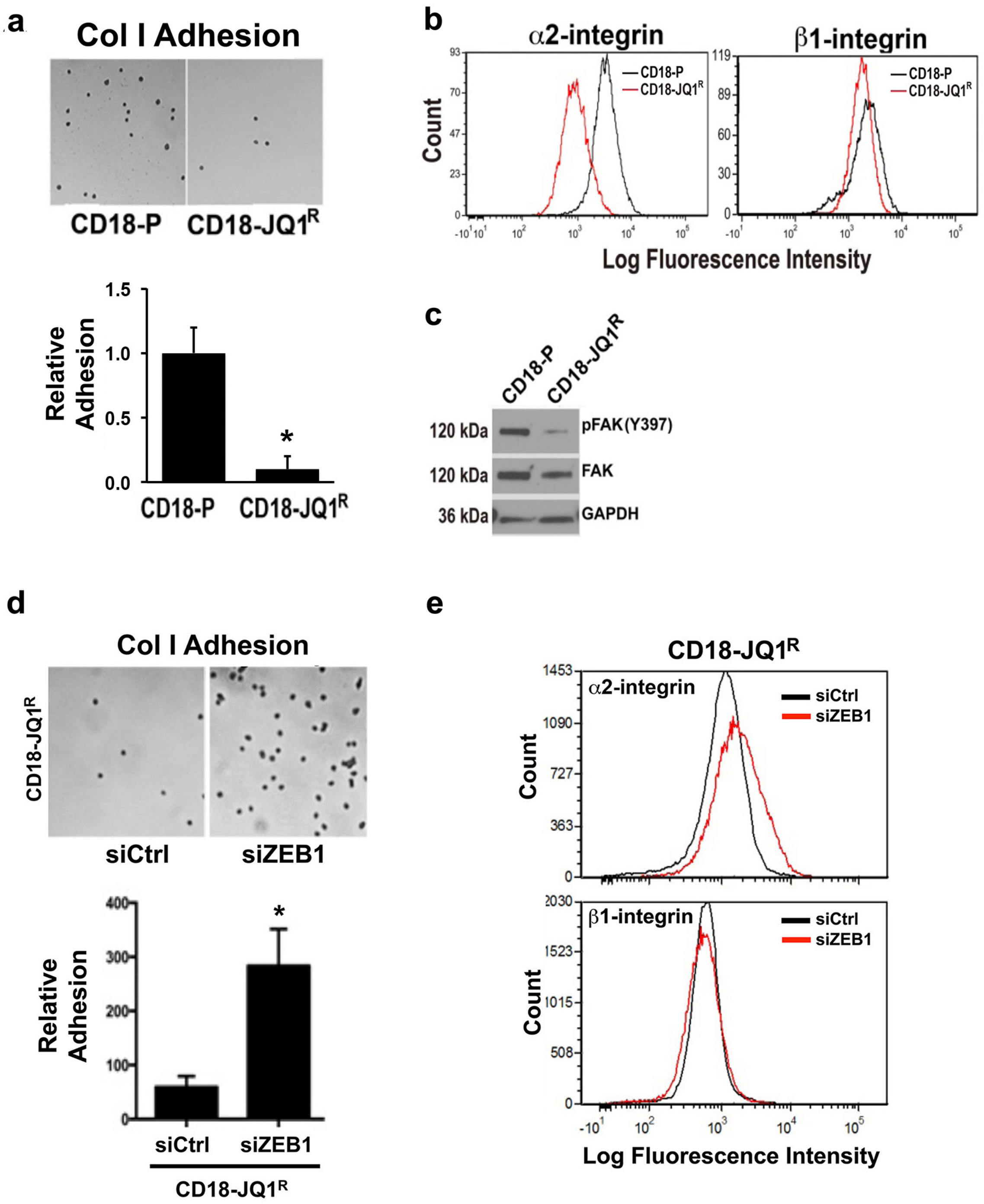
a
Col I Adhesion
CD18-P
CD18-JQ1R
Relative Adhesion
1.5
1.0
0.5
0.0
*
CD18-P
CD18-JQ1R
b
α2-integrin
β1-integrin
Count
CD18-P
CD18-JQ1R
Log Fluorescence Intensity
c
CD18-P
CD18-JQ1R
120 kDa
pFAK(Y397)
120 kDa
FAK
36 kDa
GAPDH
d
Col I Adhesion
CD18-JQ1R
siCtrl
siZEB1
Relative Adhesion
400
300
200
100
0
*
siCtrl
siZEB1
CD18-JQ1R
e
CD18-JQ1R
α2-integrin
siCtrl
siZEB1
Count
1453
1090
727
363
0
β1-integrin
siCtrl
siZEB1
Count
2030
1523
1015
508
0
Log Fluorescence Intensity
